# Small steps beyond benchmarking

**DOI:** 10.5935/0103-507X.20170022

**Published:** 2017

**Authors:** Dylan W. de Lange, Dave A. Dongelmans, Nicolette F. de Keizer

**Affiliations:** 1 Department of Intensive Care Medicine, University Medical Center, University Utrecht - The Netherlands.; 2 National Intensive Care Evaluation (NICE) Foundation - Amsterdam, The Netherlands.; 3 Department of Intensive Care Medicine, Academic Medical Center - Amsterdam, The Netherlands.; 4 Department of Medical Informatics and Public Health Research Institute, Academic Medical Center, University of Amsterdam - Amsterdam, The Netherlands.

"Benchmarking is like turning the light on!

Without benchmarking and transparency we are in the dark."

This paraphrased quote from the former president of the Institute of Healthcare Improvement Donald Berwick eloquently clarifies that we need to compare ourselves in order to optimize the outcome for our patients. In our view this is only the first step in quality improvement.

In many countries intensive care units (ICU) quality registries exist for benchmarking.^(1,2)^ The first step in the improvement of quality of care starts with measuring and comparing care structures, processes and outcome indicators with other ICUs. Turning the light on. This process identifies care structures, processes or subgroups of patients in which the outcome is not as good as the average ICU population in the benchmark. This is input for the "Plan phase" of the Plan-Do-Check-Acta (PDCA)-cycle. Obviously, many other explanations than differences in quality of care might explain these differences between ICUs.^(3)^ Differences in indicators can be caused by data quality; differences in case-mix; chance (small samples); residual confounders. Therefore, the first step is to look at the data quality. Are all participating ICUs in the benchmark actually comparing the same variables or do we use different definitions or registration methods. If we cannot agree on what we are comparing than benchmarking is useless.

Let's assume that these differences are considered to be real and not part of data quality problems, case mix differences, or chance. The following step is to identify weaknesses and solutions in the process of care (the "Do-phase" in the PDCA-cycle). Many ICUs consider this to be the most difficult part of quality improvement. Often, they do not know where to start and excuses prevail: "We have been doing this for years, so it cannot be wrong", "The solution isn't perfect, either", "No money", "Too busy", etc.

Indeed, identifying a process that can be improved with impact on the quality of care is one of the most difficult steps in quality improvement. To overcome this barrier a quality registry should support ICUs in implementing improvements by offering a "toolbox" with possible actions. Such a "toolbox" should include a list of possible bottlenecks derived from process evaluations, accompanied by a set of preferably evidence-based suggestions for concrete change.^(4)^

Another caveat is that the ambitions are too high: "We are going to be the best ICU in the country with the lowest standardized mortality ratio (SMR)!" Although this ambition is desirable the target is not very "actionable" and corrective actions are, therefore, elusive. Many of the currently available quality indicators lack the actionability and are, therefore, not useful. However, despite the fact that actionable indicators come with build-in solutions summarized in a "toolbox", implementing them in real life is cumbersome and especially enforcing them in multidisciplinary medical teams remains a challenge.^(5)^

Once, a potential improvement of a clinical process has been identified and implemented its effectiveness need to be checked (the "Check phase" in the PDCA cycle) and depending on the results new actions or new targets need to be formulated (Figure 1).

**Figure 1:**
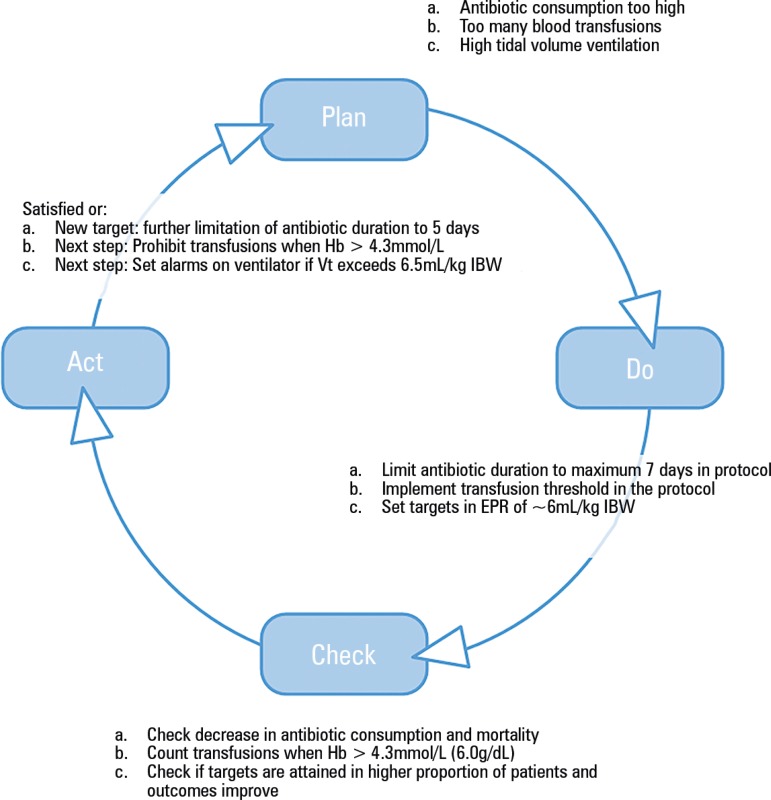
Plan-do-check-act cycle for quality improvement in the intensive care unit. Hb - hemoglobin; Vt - tidal volume; EPR - electronic patient records; IBW - ideal body weight.

## Examples of actionable indicators

A typical example of an "actionable indicator" could be the use of antibiotics on the ICU. Unnecessary long-term use of broad-spectrum antibiotics is linked to the emergence and selection of resistant bacteria, prolonged hospitalization and increased costs. Reduction of the median antibiotic duration on the ICU to 5 days is feasible.^(6)^ Such a reduction of antibiotic duration can be achieved by implementing a biomarker guided stopping of antibiotics or by a step wise reduction of antibiotic duration in comparison with peers (the benchmark). If your current practice or protocol demands 10 days of antibiotics for severe community-acquired pneumonia and the evidence advocates 5 - 7 days then the next step is to decrease the duration of antibiotics to 7 days and check your outcomes. Examples of potential improvements mentioned in the toolbox are either updating or creating of a protocol, alerts in your electronic patient records or computerized physician ordering entry whenever a prescription of more than 7 days is ordered. If mortality, days on the ventilator, and length of stay on the ICU are unchanged then a further reduction of antibiotic duration (to 5 days) can be achieved. Meanwhile, the ICU will learn that shorter courses of antibiotics are not to be feared.

Another example of an "actionable indicator" is the use of blood products.^(4)^ Many physicians feel uneasy when haemoglobin counts drop and want to transfuse such patients. Publications show similar outcomes with a more restrictive transfusion policy versus a more liberal transfusion policy.^(7,8)^ Comparing the median transfusion need in your ICU to that of the general benchmark might identify patients in which your ICU might implement a more restrictive transfusion policy without compromising outcome.^(4)^

A third example of an "actionable indicator" is the use of a low tidal volume ventilation strategy. We all know that ventilating our patients with 6 ml per kg ideal body weight tidal volume reduces the duration of mechanical ventilation and improves outcome, but adherence to these targets is poor.^(9-11)^ Yet, low tidal ventilation is truly an actionable indicator with a very clear target. If your ICU does not reach the target of low tidal volume ventilation in the subset of patients with acute respiratory distress syndrome the "toolbox" should aid in potential improvements. Applying these next steps in quality improvements represent the "Do phase" of the PDCA-cyclus.

The general idea in quality improvement is not to implement all improvements at the same time but to do it step by step. Take one (small) step at the time and compare its effect to the (national) benchmark. If it works, take the next step. Quality improvement.... do it, one small step at the time.
